# Supplementary figures and images for: Physiological and genetic characterization of heat stress effects in a common bean RIL population
Source: PLoS One. 2021 Apr 29;16(4):e0249859. doi: 10.1371/journal.pone.0249859 (PMC8084131; doi:10.1371/journal.pone.0249859)

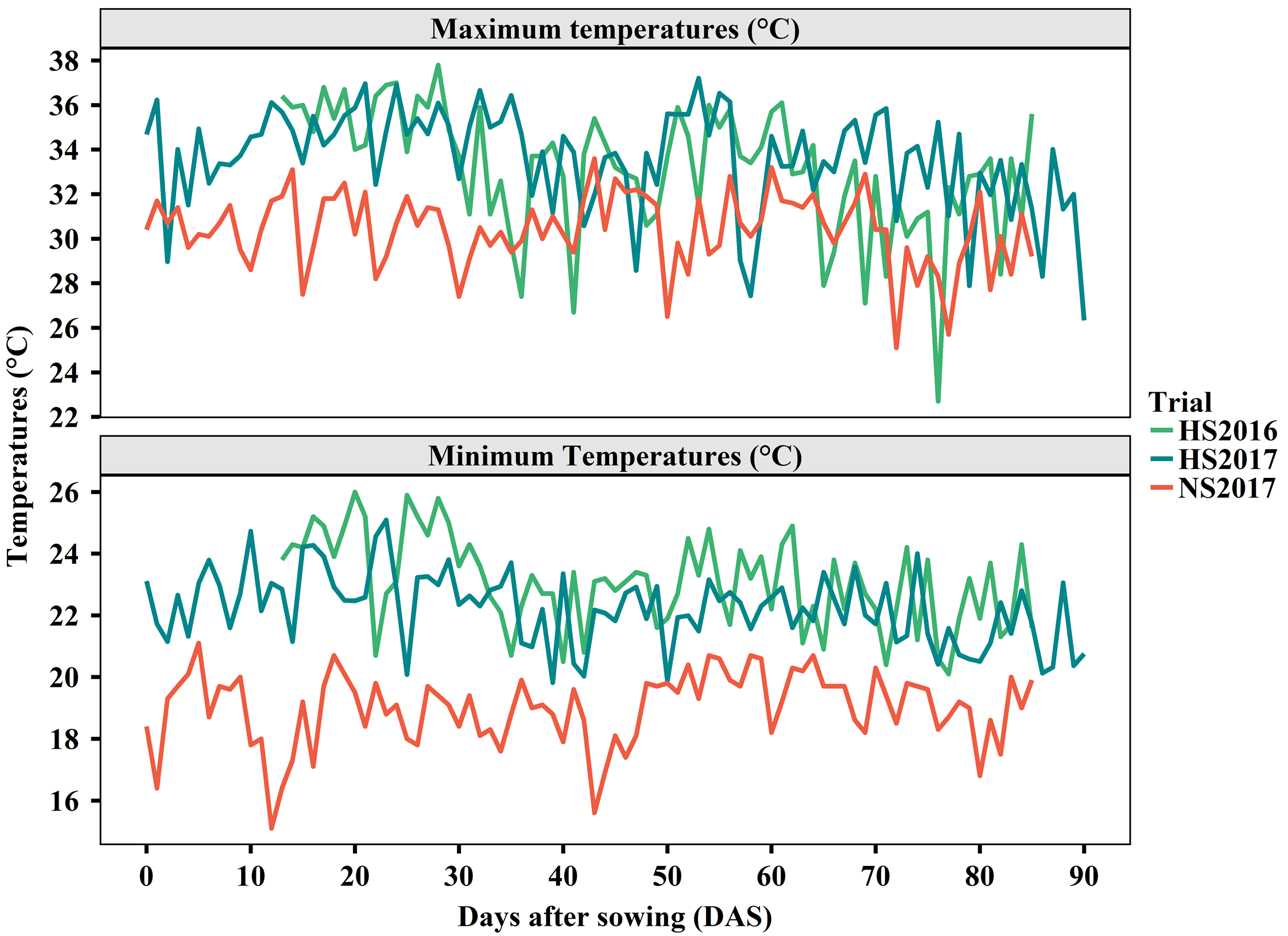

Supplement: S1 Fig — (TIF) [file pone.0249859.s001.tif]

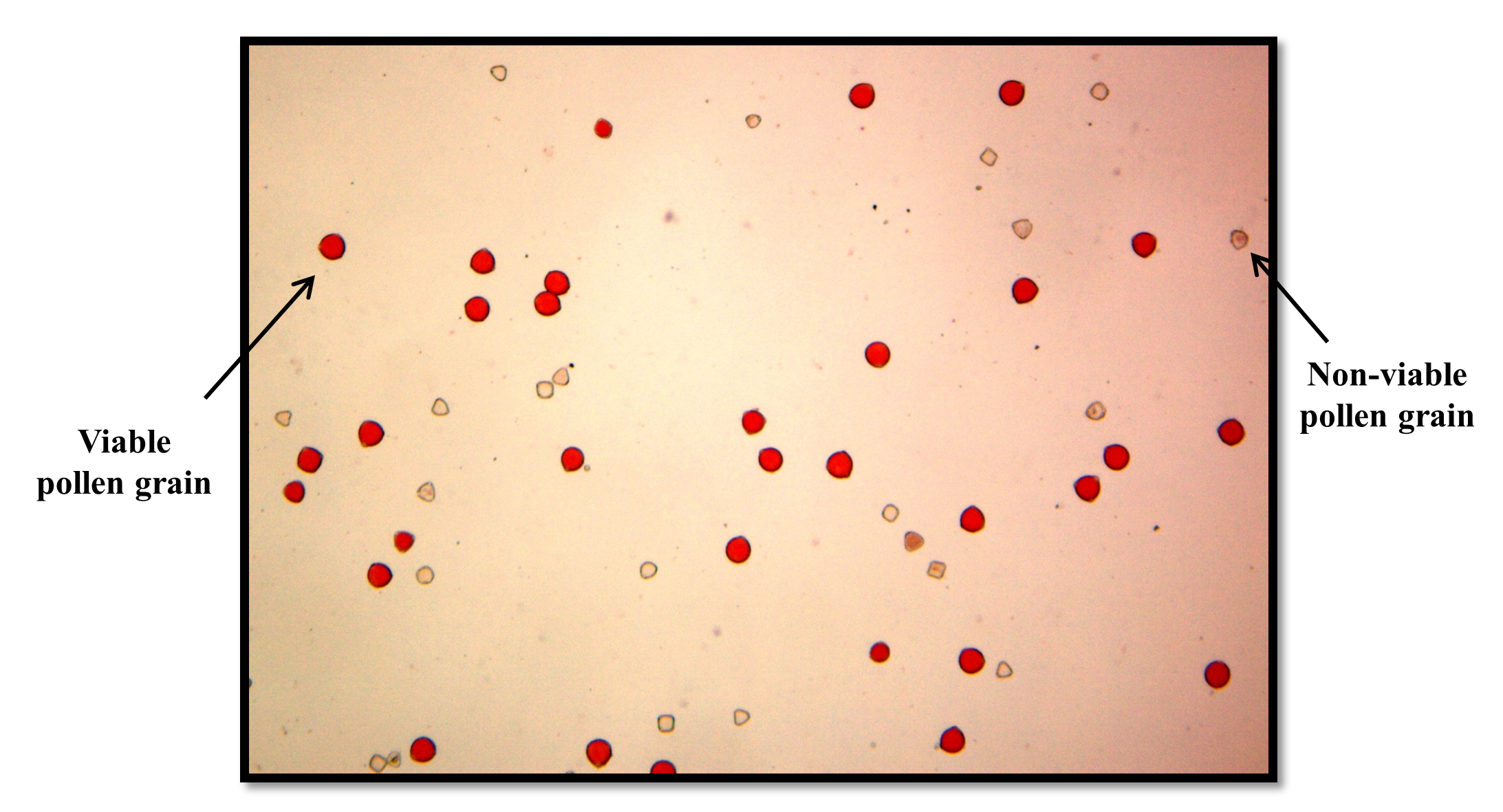

Supplement: S2 Fig — (TIF) [file pone.0249859.s002.tif]

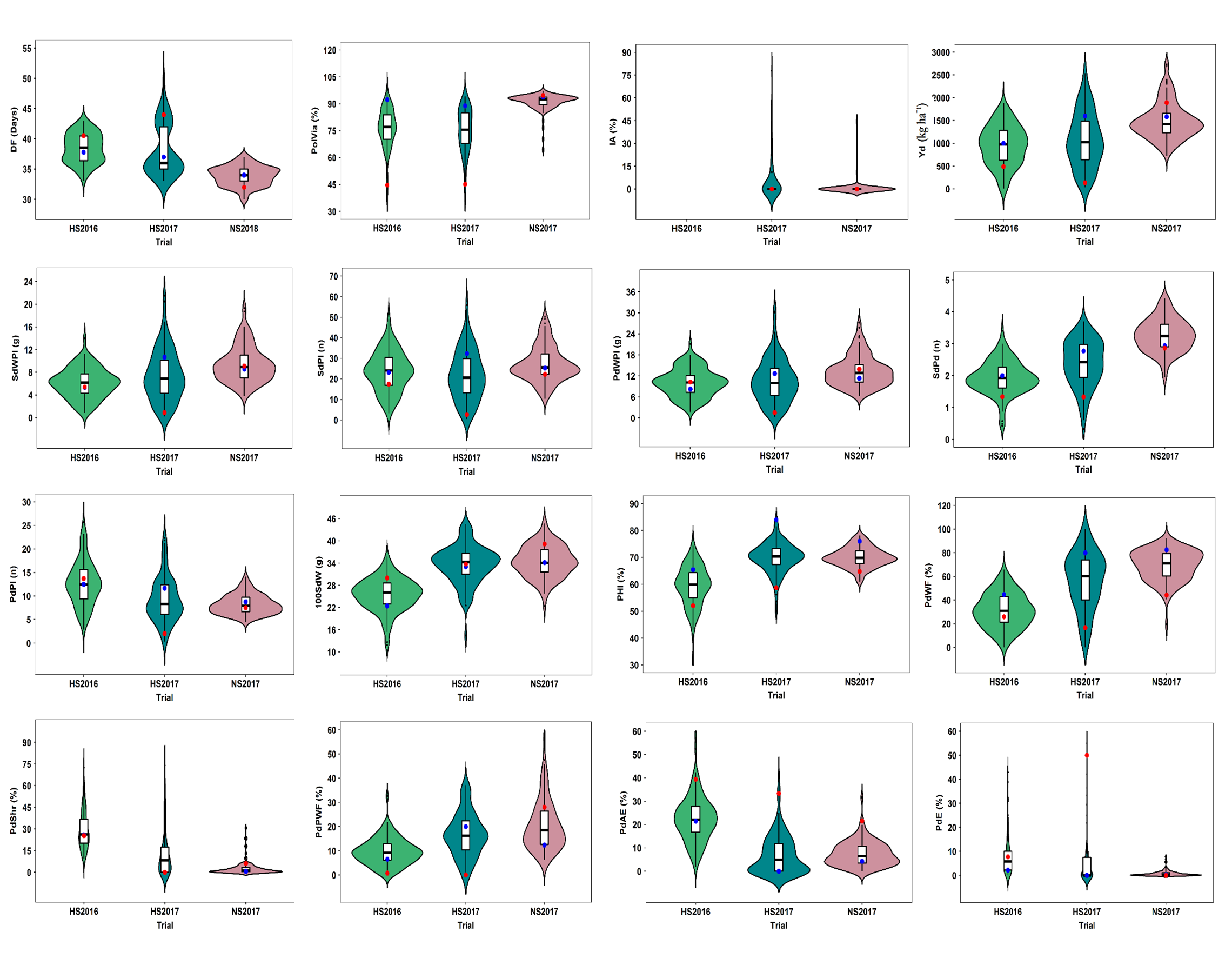

Supplement: S3 Fig — Parental lines IJR indicated as blue dot and AFR298 in red. (TIF) [file pone.0249859.s003.tif]

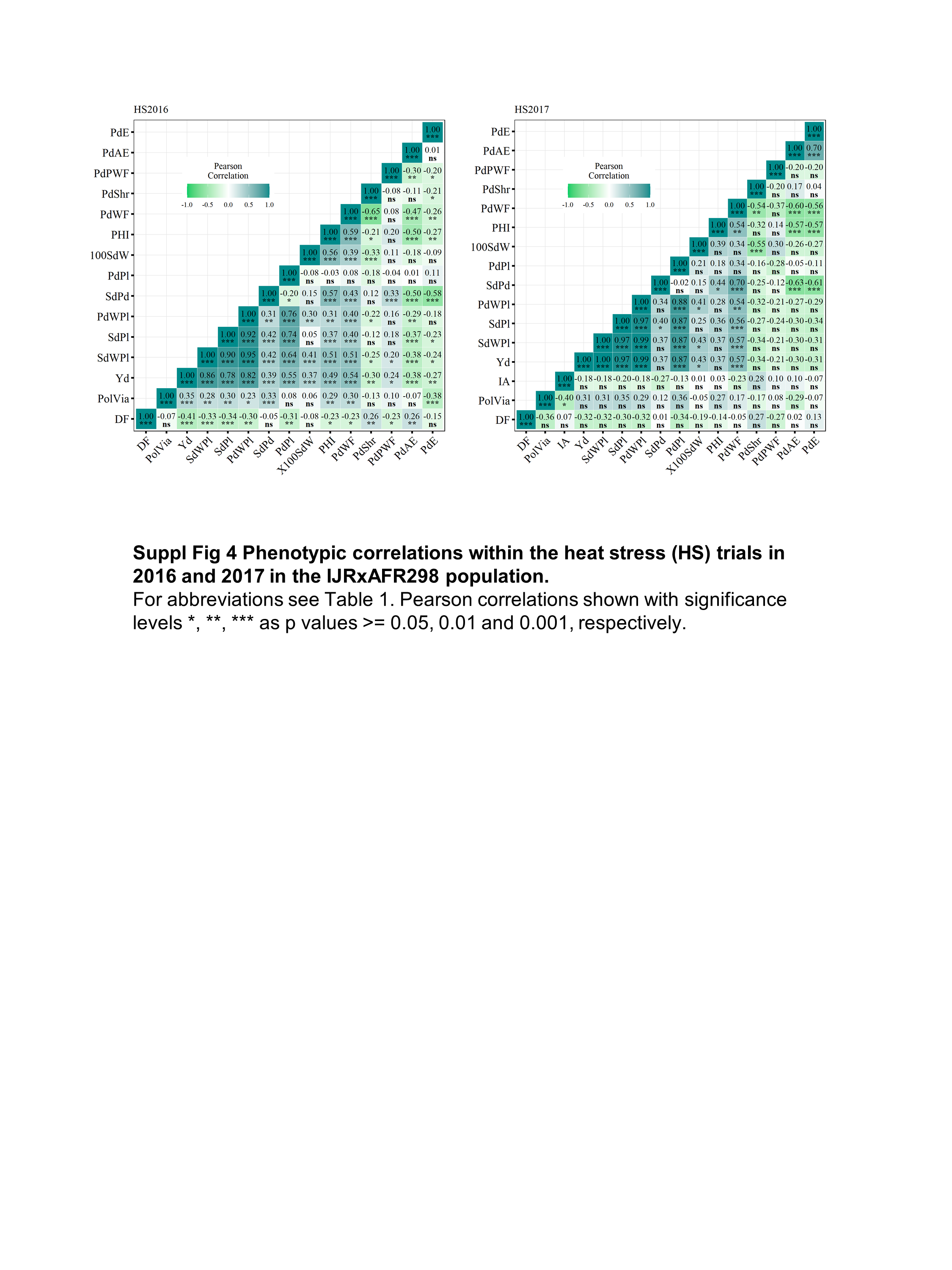

Supplement: S4 Fig — (TIF) [file pone.0249859.s004.tif]
