# Supplementary material for: Physiological and genetic characterization of heat stress effects in a common bean RIL population
Source: PLoS One. 2021 Apr 29;16(4):e0249859. doi: 10.1371/journal.pone.0249859 (PMC8084131; doi:10.1371/journal.pone.0249859)
Supplement: Attachment — Submitted filename: Reviewer Recommendation and Comments.pdf [file pone.0249859.s006.pdf]

Specific considerations:

The “Short Title” should not be equal to “Full Title".

Abstract

The Abstract should not exceed 300 words and cannot be divided into paragraphs.

L23 – Change “*Phaseolus vulgaris”* by *“Phaseolus vulgaris L.”.*

L27 - Change “RIL” by “RIL (recombinant inbred lines)”.

L27 – Change “genepool” by “gene pool”.

L37 – Change “QTL” by “QTL (quantitative trait *loci*)”.

L37 – Change “chromosome 5” by “chromosome Pv05”. Please do this for the entire manuscript.

L38 – The phrase “with the positive allele contributed by the more heat tolerant parent IJR” sounds strange.

L40 – Change “A QTL hotspot on chromosome 1 affected 6 phenology and seed formation traits” by “A QTL hotspot on chromosome Pv01 was mapped associated with six phenology and seed formation traits.

Introduction

The introduction covers the main points of the study, however, the lack of zeal in the accuracy of the references cited and in the formatting of the file is evident. The subject of QTL mapping was presented in a confusing and poorly introduced way, missing the link between the paragraphs.

According to the journal style, citations must be between “[]” and not “()”. Please review the entire file.

L51 – The reference “(5)” does not seem to support the affirmation, so it is not necessary.

L52 – Add “the” before “effects”.

L52 – The phrase “However, climatic conditions are shifting due to effects of climate change” sounds strange.

L56 – The phrase “Current research suggests that the average global temperatures have increased by ~0.8°C since 1880” need to be referenced.

L60 – Change “as” by “than”.

L63 – The introduction paragraphs must not be separated with empty lines. Please review the entire file (L72, L77, L83, L94).

L65 – The phrase “High nighttime temperatures during the reproductive phase cause heat stress in common bean, 66 and to a lesser degree, high daytime temperatures” need to be referenced.

L67 – Add “the” before “abortion”.

L70 – Add “the” before “night”.

L73/76 – I did not find any results in the reference “(16)” that supports the paragraph “Common bean genotypes of the Andean gene pool are commonly grown at mid to mid-high altitudes (1400- 2800 masl) or in cooler climates, whereas genotypes of the Mesoamerican gene pool adapt to low to mid altitude ranges (400 - 2000 masl) with higher temperatures. For this reason, Andean beans are expected to be more sensitive to high temperatures (16).”

L79 – Add references of studies carried out “under stress conditions” and “controlled environments”, separately.

L82 – Change “(Blair, Hoyos, Cajiao, & Kornegay, 2007)” by the journal style.

L84/88 – The paragraph is very confused and poorly structured. The QTL mapping makes it possible to identify loci associated with the trait of interest, in order to provide information on markers linked to the QTL that can be used for SAM. However, it is necessary to clarify that SAM and mapping are two totally different approaches. References are also missing.

L86 – Change “marker assisted” by “marker-assisted”.

L88 – Change “are” by “is”.

L90 – Change the reference by the journal style.

L92/93 – There is no point in discussing MAS if the work did not aim at MAS. It is preferable to improve the discussion of the importance of identifying markers associated with QTLs, aiming at the use of SAM.

L96 – Remove “(Indeterminate Jamaica Red)”.

L100 – Change “germplasm” by “lines”.

Materials and methods

Although a population with 107 genotypes is considered small for linkage mapping studies, it is necessary to consider the complexity of assessments for high-temperature stress. I have no experience with alpha lattice design and therefore I am not able to make considerations. However, in my opinion the NS2018 and HS2017 trials should not be considered for the study since both have no design and repetition.

L103 – Remove “(recombinant inbred lines)”.

L105 – Remove “(abbreviated from Indeterminate Jamaica Red)”.

L108 – “with wide adaptability” for what? Add the reference.

L114 – Remove “International Center of tropical agriculture”. The abbreviation CIAT has already been mentioned.

L119 – Remove “sowing at”.

L121/128 – In my opinion, that paragraph should be moved to the results section.

L122/128 – It is necessary to make it clearer that the variation of the maximum temperature mentioned, refers to the variations of the maximum temperatures of each day in relation to the total period of evaluation. The same for the minimum temperature variation.

L131,138, 145, 168 - All paragraphs begin the same "For evaluations". Rewrite.

L134 – What means “experimental plots were not replicated”? For HS2017 the 3 repetitions mentioned for HS2016 were not adopted? I did not understand.

L143 – Was the trial irrigated? If so, how many times a day? Likewise for all trials?

L145 – Add “period” or “time” after “flowering”.

L145 and L168 - In my opinion it would be better if the authors use the phrases as sub-sections (level 3). (e.g. Evaluations during flowering period / Evaluations during harvest time)

L146 - In field evaluations, where the germination of the seeds presents great variation, the most recommended is the number of days for flowering to be given by the difference in the number of days of germination and flowering.

L149 – Change “suggested” by “proposed” and correct the reference for the journal style.

L148/158 – The methodology description is extensive. The authors could cite the protocol used (Polanía et al 2016) and only highlight possible changes in the methodology.

L160 – Change the phrase by “Finally, to determine the percent viability (number of grains stained with respect to the total), the pollen grains were count using the software HYRBEAN”.

L162 – Change “read” by “evaluated”.

L166 – The caption must be more complete, adding the name of the species or "common bean", the name of the genotype used for the example and etc.

L169 – Change “variables” by “trait”,

L170 – Separate each trait on a line.

L171, 173, 174 – Change “formed” by “harvested”.

L175 – Remove “providing information on grain size.”

L195/234 – Regarding this sub-section, I can see two possible options:

1: The development of the HYRBEAN software is rewritten more succinctly and the accuracy (correlation) is just mentioned.

2: The development of the HYRBEAN software enters the objectives of the study, and the results (L229 / 234 and Fig 3) pass to the results section and are discussed later in the discussion session.

L237 – Change “y” by “and”.

L244 – The package “ggplot2” does not perform statistical calculations.

L252 – Were the F6 lines used for extraction? If yes, why was the DNA pooled?

L255 – Change “6000” by “5,398”.

L254 – Change “(1992)” by the journal style.

L259 – Remove “completely related or”.

L258/264 – The number of SNP filtered both for the polymorph of the parents and for the redundancy test can be presented in the results section.

Results

L267 – Change “Heat stress (HS)” by “HS”.

L268 – Change “non-stressed (NS)” by “NS”.

L269 – (Fig ??).

L271 – “During the reproductive phase the highest minimum daily temperatures (nighttime temperatures) were also registered in HS2016, whereas greatest maximum temperatures (daytime) were observed in HS2017.” is confused.

L274 – The graph used is very good! It clearly shows that there was a contrast between both environments (HS and NS).

279 – Change “or” by “and”.

L280/281 – One more reason not to consider HS2017 data.

The manuscript needs a review of English and formatting for the style of the journal, so I do not comment further on these errors.

L282 – “Onset of flowering was noted about 5 days later in the HS trials than in NS conditions.” Was the germination day of the plots recorded? Germination in the field

depends intrinsically on soil moisture. Please provide more information about this if the authors want to discuss the difference in the number of days for flowering.

L319/320 – The affirmation is not valid. Although most correlations are significant (4 non-significant), the highest significant correlation was 0.5 and the lowest was 0.23 (mean 0.34). These values do not support that the data are of sufficient quality to contribute to analysis.

L376 – Missing data rate less than 0.5 is very high. Usually a filter is applied around 0.8 to 0.9. Especially for Beadchip technology, where the genotyping error rate is usually very low.

L385 – Poor caption, information on the number of population genotypes, number of markers, difference between bars representing QTL and so on is lacking.

L399/401 – This belongs to the discussion.

Discussion

The discussion, besides having several formatting errors, both in the text and in the references, has several statements that need citations. In addition, based on the complexity of the trials and the magnitude of the traits evaluated, many more things could be discussed and discussed.

L444/446 – The phrase is confused.

L447/451 - Provide the reference.

L454/455 - The statement is not entirely true. It is worth mentioning that although the controlled environment does not reflect the real conditions, the evaluation in a controlled environment for a study whose main objective is "Physiological and genetic characterization" would be ideal. Field conditions being the best for selecting superior strains.

L466/467 - Due to the difference in conducting the trials, it is not possible to compare HS2016 with 2017 and conclude that nighttime temperatures have major importance in heat stress.

L504/505 - Provide the reference.

L512/513 - Provide the reference.

L515/516 - Incomplete and missing reference.

L525/526 - Repetitive, paragraph begins the same statement.

L527 – Change “QTL” by “alleles”.

References

L562 – Translate to English and update for 2018 (latest data available).

L604 – Correct the reference for “Blair MW, Hoyos A, Cajiao C, Kornegay J. Registration of Two Mid-Altitude Climbing Bean Germplasm Lines with Yellow Grain Color, MAC56 and MAC57. J. Plant Reg. 2007;1: 143-144. doi.org/10.3198/jpr2006.09.0571crg”

Supporting information

L711 – Change “y” by “and”.
